# A microscale optical implant for continuous *in vivo* monitoring of intraocular pressure

**DOI:** 10.1038/micronano.2017.57

**Published:** 2017-12-18

**Authors:** Jeong Oen Lee, Haeri Park, Juan Du, Ashwin Balakrishna, Oliver Chen, David Sretavan, Hyuck Choo

**Affiliations:** 1 Department of Medical Engineering, California Institute of Technology, Pasadena, CA 91106, USA; 2 Department of Electrical Engineering, California Institute of Technology, Pasadena, CA 91106, USA; 3 Department of Ophthalmology, University of California San Francisco, San Francisco, CA 94143, USA; 4 Department of Physiology, University of California San Francisco, San Francisco, CA 94143, USA

**Keywords:** glaucoma, intraocular pressure (IOP), microscale sensor implant, *in vivo* continuous monitoring, remote optical readout, near-infrared (NIR) broadband light, minimally invasive

## Abstract

Intraocular pressure (IOP) is a key clinical parameter in glaucoma management. However, despite the potential utility of daily measurements of IOP in the context of disease management, the necessary tools are currently lacking, and IOP is typically measured only a few times a year. Here we report on a microscale implantable sensor that could provide convenient, accurate, on-demand IOP monitoring in the home environment. When excited by broadband near-infrared (NIR) light from a tungsten bulb, the sensor’s optical cavity reflects a pressure-dependent resonance signature that can be converted to IOP. NIR light is minimally absorbed by tissue and is not perceived visually. The sensor’s nanodot-enhanced cavity allows for a 3–5 cm readout distance with an average accuracy of 0.29 mm Hg over the range of 0–40 mm Hg. Sensors were mounted onto intraocular lenses or silicone haptics and secured inside the anterior chamber in New Zealand white rabbits. Implanted sensors provided continuous *in vivo* tracking of short-term transient IOP elevations and provided continuous measurements of IOP for up to 4.5 months.

## Introduction

Glaucoma is the leading cause of irreversible blindness^1,2^, with a significant portion of patients exhibiting progressive vision loss despite treatment^3–6^. Current therapies aim to lower elevated intraocular pressure (IOP), which is the only known modifiable risk factor and thus the key parameter for clinical monitoring^7–10^. Despite its central role in glaucoma management, IOP is measured only a few times a year using specialized tonometers in the clinic. These infrequent ‘snapshot’ views of IOP are problematic because an individual’s IOP can fluctuate on a daily, weekly, or seasonal basis^11–16^. IOP can be influenced by activity, diet, and other factors that are not completely understood^17–21^. If the daily (or more frequent) IOP pattern of a glaucoma patient is available, the physician can predict disease progression and personalize therapy based on detailed knowledge of individualized trends in IOP^22^. This is similar to the approach used to treat other chronic progressive diseases such as hypertension and diabetes, in which home monitoring of blood pressure and blood sugar is integral to disease management.

In addition, pathophysiological studies and drug-discovery research demand accurate, frequent, and preferably automated assessments of IOP in humans and testing animals^23,24^. Over recent decades, animal models have significantly contributed to the understanding of the cellular and molecular bases of glaucoma^25^. However, the relationship between IOP and other factors, such as obesity, genetic contributions^26,27^, retinal ganglion cell death^28^, age, and ocular blood circulation^29^, are not fully understood yet due to the limited accuracy and usability of conventional tonometry. All tonometry approaches available in practice, such as rebounding tonometry, pneumotonometry, dynamic contour tonometry, and Goldmann applanation tonometry, perform indirect measurements of IOP. The accuracy of these approaches is adversely influenced by variations in individual corneal biomechanics^30^ and measurement complexity, rendering them unsuitable for use in large-scale animal studies. Recently developed contact-lens-based IOP sensors also provide indirect IOP measurements. They track changes in the corneal scleral angle as a surrogate measure and provide relative IOP trends in mV rather than mm Hg^31–40^. Such measurements can only be obtained for up to 24 h because of side complications that accompany long-term use^41–43^.

To overcome the aforementioned limitations, implants based on radio-frequency (RF) technologies have been used to monitor endovascular pressure^44^, intracranial pressure (ICP)^45,46^, and IOP^47–58^. The typical size of these implants ranges from a millimeter to a few centimetres. The implant’s inductive coils occupy most of this space; a larger coil is required to achieve a longer readout distance and better accuracy^52,59^. For ophthalmic implants, sensor miniaturization is important because the space available for an ocular implant is very small, especially in research animals (e.g., mice) with corneal diameters of approximately 3 mm^60,61^. Some of the RF-based IOP sensors were miniaturized down to the millimeter scale, but their practical use has been limited by short readout distances or the need for sophisticated measurement equipment (for example, spectrum, vector-network analyser) for readout^62,63^. As a result, *in vivo* measurements have thus far been obtained only with large RF implants that measure 0.5 to 1 cm in diameter^51,64,65^. Such large implants have damaged surrounding tissues and led to medical complications^66,67^. Previously investigated optical sensing approaches include a fiber-tip-based interferometry for hydrostatic pressure sensing^68–74^, a visual-identification-based method applied to pressure-sensitive microfluidic or micromechanical structures^75,76^, and laser-excited fluorescence measurements for ICP and IOP monitoring^77,78^. These approaches are promising, and with more improvements in terms of miniaturization and readout techniques, they may become practical approaches for IOP monitoring.

Here, we report an IOP-monitoring system that consists of a microscale implantable optical sensor (900 μm in diameter) and a remote optical readout detector for use in clinics, laboratories, and potentially home environments. The demonstrated advantages of our approach include: (1) microscale sizes that allow minimally invasive and safe sensor implantation in the eye using well-established intraocular lens (IOL)^79^ or silicone-haptics procedures; (2) a practical readout distance of 3 to 5 cm, which can be extended beyond 10 cm; (3) the use of non-invasive near-infrared (NIR) light from a tungsten bulb that is not perceived by the patient; and (4) excellent pressure-measurement accuracy (mean average: 0.29 mm Hg; maximum deviation: <1 mm Hg) over the clinically relevant IOP range of 0–40 mm Hg at a continuous measurement rate of 10 Hz. Using our IOP-sensing implant and readout system, we have successfully tracked artificially induced short-term transient IOP elevations in anesthetized rabbits for a period of 1 h. We also tracked long-term changes in IOP in awake rabbits over the course of 4.5 months. Our sensor measurements were verified using readouts concurrently obtained using the Tonovet (Icare, Vanda, Finland), and comparison between the measurement sets revealed good consistency.

## Materials and methods

### A microscale nanodot-enhanced IOP sensor and principles of operation

**Figure 1:**
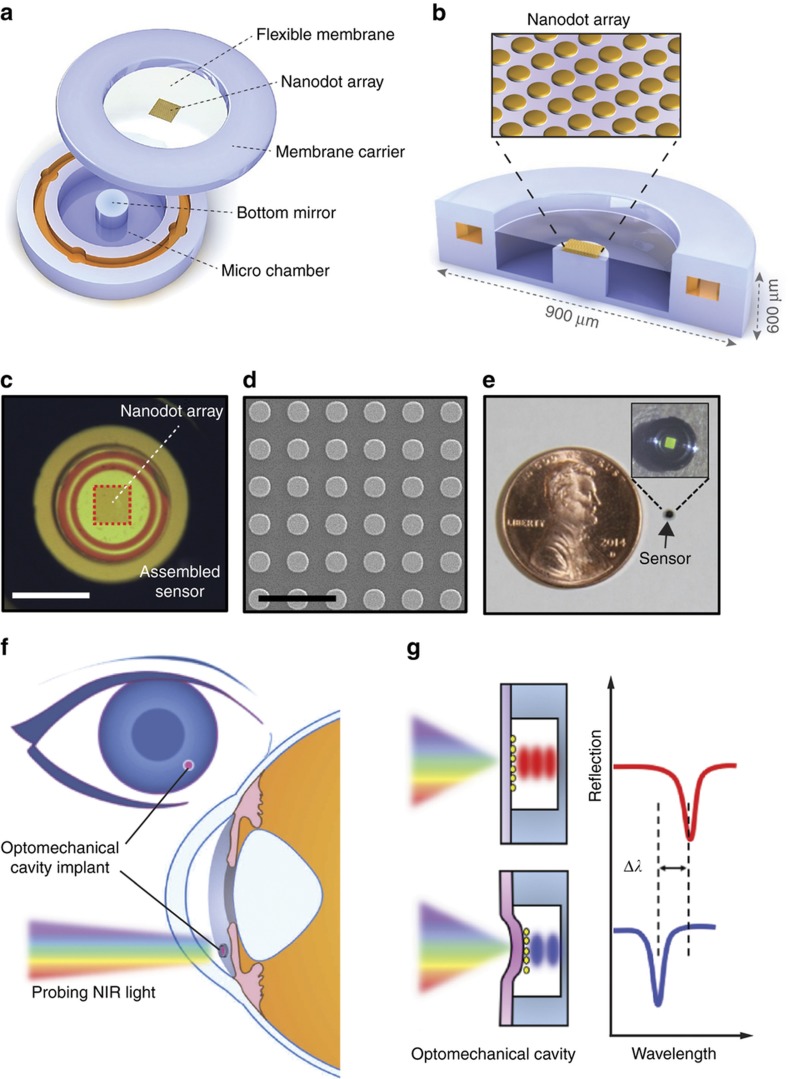
A microscale nanodot-enhanced intraocular pressure (IOP) sensor and operating principles. (**a**) A three-dimensional (3D) illustration of the top structure with a nanodot-embedded deformable SiN membrane and the bottom structure with a Si reflective surface in the center and a cylindrical hollow cavity, before assembly. (**b**) A cross-sectional schematic view of an assembled sensor and a zoomed-in image of the nanodot array in the SiN membrane (inset). (**c**) A microscope image taken perpendicular to the device surface showing the square nanodot array in the middle of the sensor (scale bar: 500 μm). (**d**) Scanning electron microscopy (SEM) image of the gold nanodot array on a SiN membrane: the diameter of each dot is 600 nm (scale bar: 2 μm). (**e**) A photograph of a completed device with a diameter of 900 μm (inset: a zoomed-in image of the sensor taken at an angle at which the rectangular nanodot array assumes a green-blue color). (**f**) A nanodot-enhanced IOP sensor located in the anterior chamber and interrogated using NIR light. (**g**) A schematic illustrating the shift in the sensor-reflected resonance as a function of the gap distance within the sensor’s optical cavity, which in turn is related to IOP.

The implantable IOP sensor is a hermetically sealed hollow disk that serves as a pressure-sensitive optical resonant cavity. It consists of a top half that is in the form of a micromachined silicon ring with a deformable silicon-nitride (SiN) membrane and a bottom half that contains a solid reflective Si surface that serves as a mirror (Figures 1a and b).

The two halves are assembled together using medical grade epoxy, resulting in a 7-μm gap between the SiN membrane and the Si mirror that forms the optical cavity. The optical resonance of the cavity is further enhanced by the placement of a gold nanodot array fabricated onto the deformable SiN membrane (Figures 1b–d). The gold nanodot array optimizes the reflectivity of the top SiN membrane to match the reflectivity of the bottom silicon surface and maximizes the amplitude of the optical resonance. The nanodot array dimensions for optimal membrane reflectivity were identified using parametric finite-difference-time-domain (FDTD) simulations and experimental measurements (Supplementary Figure S1). Based on this analysis, a 200×200 μm^2^ array with a dot diameter of 600 nm and dot-to-dot pitch of 1000 nm (Figures 1c and d) was used to double the amplitude of the cavity resonance. The final sensor dimensions after assembly are 900 μm in diameter and 600 μm in height, thus the area of the assembled sensor including the surrounding Si frame is 0.635 mm^2^ and the volume is 0.381 mm^3^ (Figure 1e). Moreover, the area of the core optomechanical cavity is 0.283 mm^2^ (600 μm in diameter) occupying only 44% of the entire sensor area and suggesting further miniaturization. This compact size is an order of magnitude smaller than the state-of-the-art research devices^46,62,63^ and three orders of magnitude smaller than commercially available sensors^65^.

For IOP monitoring, the sensor is implanted into the eye where its deformable SiN membrane is exposed to the IOP and interrogated using the broadband invisible NIR regime (800–1100 nm) of a tungsten light bulb (Figure 1f) (see *in vivo* testing below for details on the sensor implantation). At a given IOP, the sensor has an associated gap size and reflects a characteristic resonance that will have a spectral signature or a spectrum consisting of reflectance extrema, that is, peaks and dips, at specific wavelengths (Figure 1g, top). When the IOP increases, the flexible SiN membrane is deflected inwards and causes the gap to decrease, and consequently this results in a new resonance spectral signature consisting of reflectance dips that are shifted to shorter wavelengths (Figure 1g, bottom). When the IOP decreases, the SiN membrane deflects outwards and causes the gap to increase, and the new resonance spectrum will consist of reflectance dips that are shifted to longer wavelengths. The location of these resonance peaks and dips can be identified using a commercially available mini-spectrometer and used to determine the gap separation and therefore the ambient pressure, that is, the IOP.

### Design of the nanodot-enhanced IOP sensor

We chose the near infrared (NIR) wavelength range of 800–1100 nm as a broadband light source to take advantage of the fact that NIR light is minimally absorbed in the cornea and the aqueous humor and is also invisible to the human eye.

SiN was selected as the material for the deformable membrane due to its high optical transparency, large refractive index, extremely low water permeability, and robust mechanical resilience. The relatively high refractive index of 1.98 for SiN allows the implementation of a sensor cavity with a strong optical resonance in saline or aqueous humor because the resonance amplitude of an optical cavity is heavily dependent on the reflectivity of its surfaces. If the reflectivity is too high or too low, the resonance may become very sharp or flat, which significantly lowers the SNR ^80–82^.

The thickness of the SiN membrane was optimized to maximize the amplitude of the sensor resonance. In preliminary studies, we observed that the detected sensor-resonance spectrum was the result of two separate resonances: one from the thickness of the silicon-nitride membrane, and the other from the cavity gap between the nitride membrane and the bottom silicon surface. The resonance from the silicon nitride membrane defines the outer low-frequency envelope while that of the cavity forms the inner higher frequency component (Supplementary Figure S2). Hence, using FDTD simulation, we determined that the optimal thickness of the silicon-nitride membrane was 0.3 μm, which allowed the maximum of the outer low-frequency envelope to be centered in the 800–1100 nm range.

The optimal diameter for the 0.3 μm-thick SiN membrane was determined using a series of finite-element-method (FEM) simulations and a high-order analytical model for a thin diaphragm (Supplementary Figure S3)^83,84^. For the pressure range between 0 and 40 mm Hg, SiN membranes with diameters between 200 and 1000 μm were all found to exhibit linear deformation as a response to hydrostatic pressure changes. For the present proof-of-concept study, 600 μm was conservatively chosen as the SiN-membrane diameter to facilitate the fabrication and assembly requirements.

The dimensions of a gold-nanodot array for optimal membrane reflectivity were identified using a series of finite-difference-time-domain (FDTD) simulation and experimental measurements^84^. The use of an optimized 200×200-μm^2^ array with a dot diameter of 600 nm and dot-to-dot pitch of 1000 nm doubles the amplitude of the cavity resonance. And, the cavity gap was set at 7 μm to obtain approximately three peaks and three valleys in the reflection spectra. The presence of multiple peaks and valleys in a single spectrum improves the accuracy of the IOP determination.

### IOP-sensor fabrication

The top half of the sensor with a SiN membrane was created by growing 2 μm-thick thermal silicon dioxide (SiO_2_) and a 300 nm-thick silicon-nitride (SiN) layer using a low pressure chemical vapor-deposition technique in sequence on a double-side-polished wafer (diameter: 4 inches; thickness: 300 μm). The SiN and SiO_2_ layers on one side of the wafer were completely removed using reactive ion etching, followed by buffered hydrofluoric (BHF) acid.

The hollow circular opening in the top structures was created by first depositing a 300-nm-thick Al_2_O_3_ layer in an e-beam evaporator (Mark 50 System, CHA Industries, Inc., Fremont, CA, USA) onto the cleaned side of the wafer. Photolithography (photoresist: AZ 9245, AZ Electronic Materials, USA Corp., Branchburg, NJ, USA) was then used to define solid disks in the Al_2_O_3_ layer as masks, and Bosch deep reactive ion etching (Plasmalab System 100 RIE/ICP, Oxford Instruments, Abingdon, UK) was performed through the wafer at an etch rate of 1–2 μm per min, stopping at the SiO_2_ layer at the bottom surface of the wafer.

The gold-nanodot arrays were placed on the SiN membrane by first soaking the top structure wafer in BHF to remove the SiO_2_ layer, and electron-beam lithography (Raith EBPG 5000+ Electron Beam Writer, Raith Nanofabrication, Dortmund, Germany) was then performed to pattern the gold nanodots on the suspended SiN membrane. To separate out each individual top structure from the processed wafer, another run of the reactive ion-etching (RIE) process was used as a dicing step.

The bottom structure of the sensor, which consists of a reflective Si surface and a hollow cylindrical cavity, was fabricated in another Si wafer. A carefully controlled Bosch process was used to create 7 μm-deep cylindrical trenches with a smooth bottom surface (AFM-measured peak-to-peak roughness: 1.3 nm) to serve as the mirror in the sensor cavity. Finally, the top and bottom structures were aligned under a microscope and bonded together using a medical grade epoxy. All sensors were pre-characterized in a pressurized chamber after fabrication and devices with high SNR values (>15 dB) were selected for implantation.

## Results

### Sensor characterization in a controlled pressure chamber

**Figure 2:**
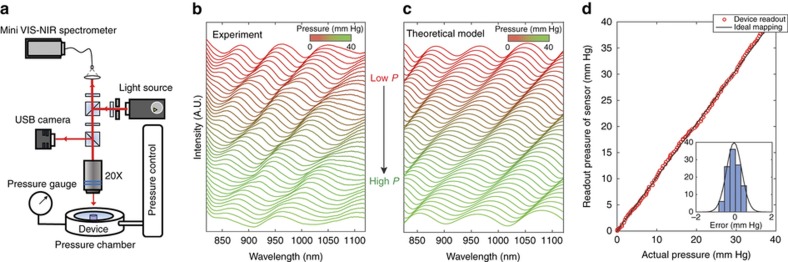
Sensor characterization in a controlled pressure chamber. (**a**) Intraocular pressure (IOP) sensor characterization in a controlled pressure chamber with a digital pressure gauge. A schematic of the sensor optical resonance detector is shown above the pressure chamber. (**b**) Experimentally determined spectra from the sensor in the pressure range from 1 to 40 mm Hg with the spectra corresponding to 1 mm Hg shown at the top and the spectra for 40 mm Hg shown at the bottom. The spectra for the intervening IOPs are shown in sequence from top to bottom. (**c**) Theoretically predicted spectra corresponding to the pressure range from 1 (top spectra) to 40 mm Hg (bottom spectra). (**d**) Highly linear, very close one-to-one matching between the sensor measurements (vertical axis) and the digital pressure-gauge readouts (horizontal axis). The black line shows a theoretical perfect match of sensor and digital pressure-gauge readings, and the red circles indicate actual experimental measurements corresponding to the pressure readout based on the optomechanical model (OMM). Histogram shows the error distribution (RMSE: 0.29 mm Hg). Even in the worst case, the sensor reading was within ±1 mm Hg of the digital pressure-gauge readings.

The accuracy, range, and linearity of the fabricated nanodot-enhanced sensors were tested in a controlled pressure chamber filled with saline (Figure 2a). We varied the pressure inside the chamber between 0 to 100 mm Hg at steps of 0.05 mm Hg using an integrated water column and a programmable syringe pump (NE 1000, ABC Scientific, CA, USA). We monitored the pressure using an electronic pressure sensor (1210 Pressure Sensor, TE Connectivity Ltd., Schaffhausen, Switzerland) with an accuracy of ±0.1 mm Hg. A custom-built table-top detection system (Figure 2a; See section “Custom-Built Optical Detector” in Supplementary Information) was used to excite the sensor’s optical cavity with broadband NIR light (800–1100 nm) through an optically transparent window located in the lid of the chamber. The reflected optical resonance spectra from the sensor were then collected using a commercially available mini-spectrometer (MAYA 2000 Pro, Ocean Optics, Dunedin, FL, USA) that was connected to the detection system.

As expected, the reflectance dips in the spectra of the sensor showed a systematic shift to shorter wavelengths as the gap of the sensor cavity decreased with increasing hydrostatic pressure (Figure 2b). The experimentally measured spectral shifts (Figure 2b) matched well with the theoretically predicted spectra (Figure 2c). The analytical prediction was first performed by an optomechanical model (OMM) that consisted of physical and material parameters, enabling the calculation of ambient pressure for any given spectrum measurement (See Supplementary S4 and S5). The pressure calculation relied on the fact that each pressure (or each cavity gap) produced multiple resonance peaks with unique locations and adjacent spacing that allowed us to achieve one-to-one mapping between the measured spectrum and its corresponding IOP^85^. In the pressure range from 0 to 40 mm Hg, the nanodot-enhanced sensor showed highly linear responses, and the deviations from the electronic pressure-gauge reading remained below 1 mm Hg with root mean square error (RMSE) of 0.29 mm Hg (Figure 2d).

### Sensor characterization in *ex vivo* rabbit eyes

**Figure 3:**
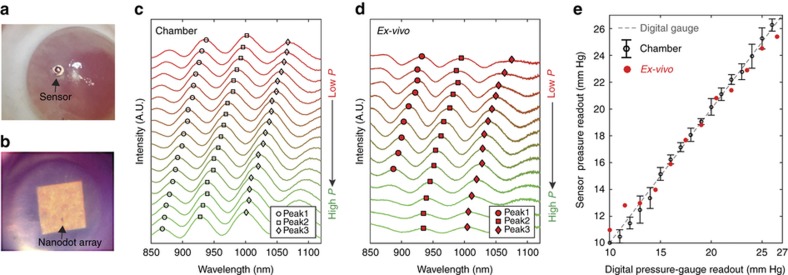
Sensors in *ex* *vivo* rabbit eyes and spectra collected over a range of induced intraocular pressures (IOPs). (**a**) A photograph of an implanted sensor inside the anterior chamber of an *ex vivo* rabbit eye. (**b**) A microscope image showing the 200-μm^2^ nanodot array of a sensor taken thorough the cornea and the aqueous humor in an *ex vivo* eye. (**c**) The spectra from a sensor tested in the controlled pressure chamber for pressures. The open symbols show the location of the three reflected resonance peaks. (**d**) The spectra from the same sensor shown in **c** when tested in an *ex vivo* eye as the internal pressure of the *ex* *vivo* eye was varied using a 21-gauge needle and a syringe pump. Red symbols show the location of the three reflected resonance peaks. (**e**) Mapping comparison between the sensor’s pressure readout obtained from pressure chamber testing (black circle) and *ex vivo* eye testing (red circle). The vertical axis indicates sensor measurements and the horizontal axis indicates the digital pressure-gauge readouts. The black error bars indicate standard deviations (*n*=15).

The sensors that were evaluated in the controlled pressure chamber were next characterized in *ex vivo* rabbit eyes. The sensors were inserted through a clear corneal incision and placed directly onto the iris with the SiN-membrane side facing up. (Figures 3a and b).

The IOP was then systematically increased via saline infusion at steps of 1 mm Hg. The resulting IOP was monitored using a 21-gauge needle that was directly inserted into the anterior chamber and connected to the same digital pressure gauge that has been used for the characterizations in the controlled pressure chamber. In parallel, the resonance spectral signatures of the sensor were collected using the previously described detector system. Figures 3c and d show the spectral measurements made for the same sensor in the controlled pressure chamber and in an *ex vivo* rabbit eye. The extrema locations from both the test chamber characterizations (Figure 3c) and the *ex vivo* eye characterizations (Figure 3d) for the same sensor are very consistent with one another. The differences between the readouts from the same nanodot-enhanced sensor implanted inside an *ex vivo* eye and the concurrently obtained readouts from the digital pressure gauge connected to the same *ex vivo* eye were less than 1.3 mm Hg in all cases (Figure 3e).

### Sensor performance in rabbit eyes *in vivo*

**Figure 4:**
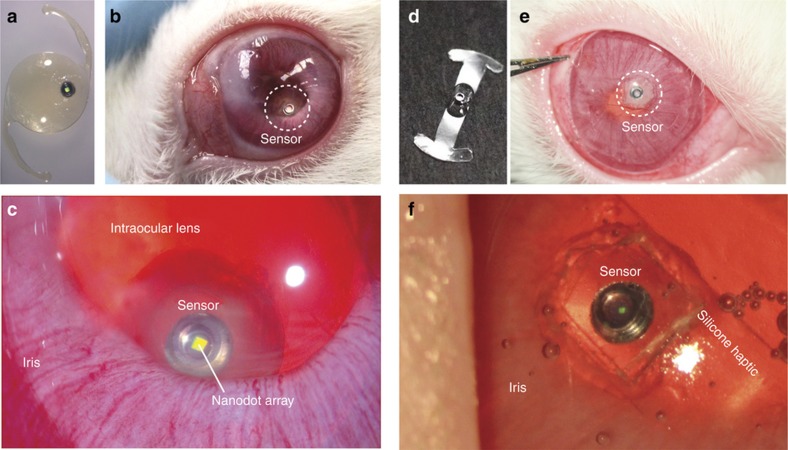
Nanodot-enhanced IOP sensors in rabbit eyes. (**a**) An IOP sensor attached to a one-piece acrylic IOL. The sensor is the black circular object with a central bright area that represents the nanodot array. (**b**) A nanodot-enhanced IOP sensor (located within the dashed circle) implanted into a rabbit eye. (**c**) A higher magnification view of the same sensor as in **b**. The bright square in the middle of the sensor represents the nanodot array. The translucent material surrounding the sensor is the epoxy adhesive used in the sensor assembly and sensor attachment to the IOL. (**d**) A nanodot-enhanced sensor attached to thin silicone membrane haptics. (**e**) A nanodot-enhanced sensor mounted on silicone haptics (located within the dashed circle) implanted into a rabbit eye. (**f**) Higher magnification of the same sensor as in **e**. The bright square in the middle of the sensor represents the nanodot array. The silicone haptics appear transparent after implantation into the eye.

Sensors whose performance metrics had been fully characterized in the controlled pressure chamber were implanted into the eyes of New Zealand white rabbits to investigate their performance *in vivo*. Two methods were used for sensor implantation. In one method, individual sensors were attached to an intraocular lens (IOL) (Figure 4a) and placed into the lens capsular bag following the surgical extraction (See section “Surgical Procedures” in Supplementary Information) of the crystalline lens (Figures 4b and c; Supplementary Video 1). A cavity of approximately 1 mm in diameter and 0.5 mm in depth was mechanically cut into the IOL. Sensors were placed into this cavity using medical grade UV adhesive (Loctite 3321, Loctite Corp. Rocky Hill, CT, USA) with the deformable membrane side facing outwards from the IOL cavity. In the other method, sensors were attached to 125-μm-thick clear silicone-membrane haptics (Figure 4d), and rolled up for insertion into the anterior chamber through a clear corneal incision. The silicone haptics were manually fabricated from medical grade silicone membrane (125 μm thick) (BioPlexus, Ventura, CA, USA) into a barbell shape approximately 2.5 mm in width and 12.5 mm in length. The two haptic arms then spontaneously unfolded to extend into the iridocorneal angle and mechanically anchor the sensor within the anterior chamber (Figures 4e and f; Supplementary Video 2).

**Figure 5:**
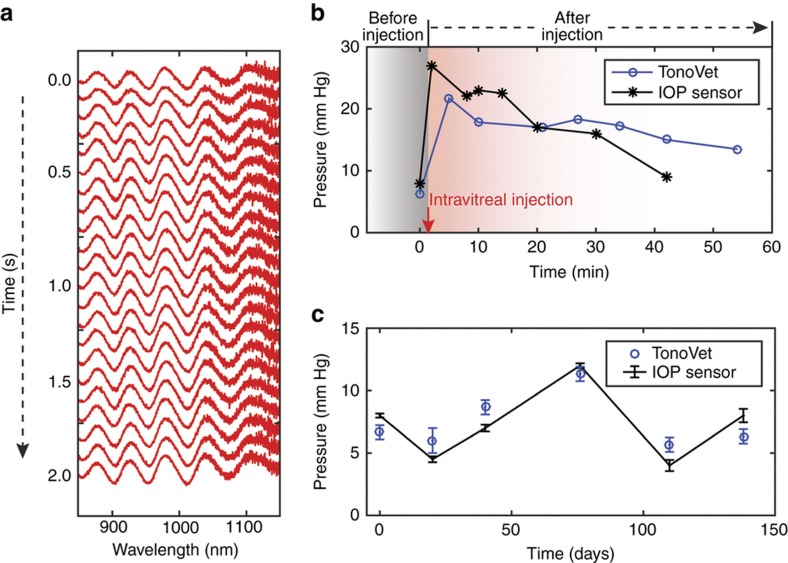
*In vivo* spectra and IOP measurements. (**a**) A 2 s block of stacked resonance spectra from a 20 s remote optical detector measurement session performed at 10 Hz. The first spectra obtained at the start of the 2 s block are displayed at the top of the stack. The spectra recorded at subsequent times are sequentially stacked towards the bottom. (**b**) The IOP measurements before and after intravitreal injection of hypertonic saline to cause transient IOP elevation at 138 days after implantation. The sensor-derived IOPs (black symbol, *n*=20) and the TonoVet-derived IOPs (open blue circles, *n*=6) mirror each other and show a rise in IOP after injection followed by a gradual decline over the following 43–55 min. Error bars indicate standard deviations. (**c**) A comparison of IOP derived from sensor measurements *vs* TonoVet IOP measurements over a 138-day period.

Next, we characterized the *in vivo* performance of implanted sensors in rabbit eyes using the remote optical detector. The data reported here were obtained from five sensors implanted in five rabbits (or one sensor per rabbit) for up to 4.5 months. The optical alignment was carried out by monitoring both camera image and the spectra in real time. The initial alignment between the sensor and the detector was achieved relatively easily by using a co-axially integrated USB-camera and maximizing the reflection back from the sensor until the sensor appeared as a bright spot saturated with light in the image. We know from the bench-top testing that the bright spot guarantees an optical alignment within ±3° (Supplementary Figure S8). Then the alignment was further improved by manipulating the mini-translational stages of the detector until the resonance amplitude captured by the spectrometer appeared maximized. This would lead to an alignment accuracy within ±1°. Furthermore, in order to reduce the potential error in angular alignment during *in vivo* measurements, we continuously record the spectra at a sampling rate of 10 Hz and choose only the highest-quality spectra with SNR over 15 dB. Using this high-speed sampling approach allowed us to filter out erroneous measurements with low SNRs in post-measurement processing and to minimize the IOP-estimation error that could results from movements (such as breathing) of the test subject. During each recording session, the resonance spectra from the sensor were recorded at a rate of 10 Hz for 20 s. Examples of 20 individual resonance spectra collected over 2 s from a sensor following the *in vivo* implantation are shown in Figure 5a. The captured resonance signals were stable, with an excellent signal-to-noise ratio (SNR) of 15 dB, which was close to 16 dB measurement that had been observed during pressure-chamber characterization. The deterioration in SNR above 1050 nm originated from the decreased sensitivity of the silicon detector used in the spectrometer. IOP measurements were also performed concurrently using a commercial rebound tonometer (TonoVet, Icare, Vanda, Finland).

The ability of nanodot-enhanced sensors to report the short-term increases and decreases in IOP *in vivo* was tested on anesthetized rabbits. We injected 5% hypertonic saline intravitreally into the eyes with the implanted sensors to cause a quick transient increase in IOP to a peak of 25–30 mm Hg followed by a gradual return to the baseline over approximately 1.5–2 h. The resonance spectra of the sensors were captured in parallel with IOP measurements using the TonoVet for 45–60 min after these injections. An example of the experimentally induced IOP profile measured by the nanodot-enhanced sensor and by the TonoVet is shown in Figure 5b. Prior to the saline injection, the sensor-recorded IOP was 8 mm Hg and the TonoVet-derived IOP was 6.3 mm Hg. The first sensor-recorded IOP captured at approximately 2 min after the intravitreal saline injection showed an increase in IOP to 27.0 mm Hg (see also Supplementary Figure S6), which was followed by a gradual decrease over 43 min. This mirrored the IOP changes recorded by the TonoVet (Figure 5b).

Moreover, the IOPs from nanodot-enhanced sensors have been measured for up to 138 days after the *in vivo* implantation. (138 days represents the last data point obtained prior to the submission of this manuscript). Figure 5c shows six IOP measurements obtained from an implanted sensor and six concurrent IOP measurements obtained using the TonoVet on Days 0, 20, 40, 76, 110, and 138 after implantation. IOP readings from the sensor on each day were averaged from 200 measurements made over 20 s: these were 7.8±0.16 (Day 0), 5.3±0.24 (Day 20), 7.7±0.27 (Day 40), 12.3±0.19 (Day 76), 3.9±0.45 (Day 110), and 6.6±0.54 (Day 138) mm Hg. The corresponding TonoVet IOP readings were 6.7±0.6 (Day 0), 6.0±1.0 (Day 20), 8.7±0.6 (Day 40), 11.3±0.6 (Day 76), 5.7±0.6 (Day 110) and 6.3±0.6 (Day 138) mm Hg. Each TonoVet IOP value is an average of 3–6 individual measurements.

## Discussion

The present study has provided proof of concept for the remote detection of IOP from an implanted nanodot-enhanced IOP sensor using invisible NIR light. The sensor is extremely compact and measures less than 1 mm in size. The bench testing in a controlled pressure chamber demonstrated IOP readings in the clinically useful range of 0–40 mm Hg with an average accuracy of 0.29 mm Hg and in the *ex vivo* eyes with an accuracy of ±1.3 mm Hg when measured against a commercial pressure gauge in both cases. In our algorithm, the locations of the extrema (that is, peaks or valleys) determine the pressure. Therefore, when one of the extrema completely shifts out of the measurement-spectrum window, the magnitude of the inaccuracy suddenly increases. If we avoid a shift-out by limiting the measurement range to, for example, between 10 and 11 mm Hg, the sensor provided a much-improved pressure resolution of 0.07 mm Hg (Supplementary Figure S7). Such a fine level of pressure resolution easily exceeds the 1-mm Hg resolution observed in commercial tonometers and is very promising. *In vivo* testing showed that implanted sensors were able to reliably measure short-term increases and decreases in IOP that matched concurrently obtained TonoVet IOP readings. Furthermore, the sensor performance was also confirmed over a period of 138 days during which the sensor’s IOP readings paralleled those obtained using the TonoVet. The remote optical detector also has a simple configuration that allows easy optical alignment for use in a laboratory and clinics, and it can be further simplified and implemented in a form of a hand-held detector for use in the home environment.

The results from sensor testing in *ex vivo* eyes showed that IOP readings of the sensor differed from IOPs determined by the commercial pressure gauge by ±1.3 mm Hg (Figure 3d). This accuracy improves to ±0.29 mm Hg over the 0–40 mm Hg range when the same sensor was characterized in the controlled pressure chamber using the commercial pressure gauge (Figure 2d). In *ex vivo* eye testing, the pressure gauge was connected to the inflow line, and the inflow line tip was inserted through an entry point into the eye. As a result, the pressures sensed by the pressure gauge and the sensor in the eye may be slightly different due to several factors including the difficulty in establishing a tight seal around the infusion tubing at its insertion point into the *ex vivo* eye for an extended period of time, and the challenge of maintaining a stable pressure level given the intact outflow pathways in an *ex vivo* eye.

*In vivo* IOP readings from the sensors were all compared to those obtained using the TonoVet rebound tonometer to avoid repeated use of invasive manometric determinations of IOP. As the TonoVet is a hand-held tonometer, its readings are affected by inherent variations in positioning and by the corneal surface hydration state of the anesthetized rabbits during the recording session. Nevertheless, all but one sensor reading were within the 2-mm Hg error range of the concurrently performed TonoVet readings. (The manufacturer-specified accuracy of the TonoVet utilized in our studies was ±2 mm Hg.) Note that the TonoVet is not specifically calibrated by the manufacturer for rabbit corneal biomechanical properties, and its IOP readings from rabbits are known to be systematically lower than intraocularly determined manometric IOPs^86^. More precise and detailed characterization of our sensor’s *in vivo* performance in subsequent work will require improved reference-IOP measurements in the eye.

Our system’s temporal resolution is 10 Hz, and capturing a single spectrum necessary for an IOP measurement requires only 0.1 s. This implies that the system can easily detect changes in IOP in less than 1 s. The 2-second blocks of continuous IOP data (measured at 10 Hz) in Figure 5a showed that the nanodot-enhanced IOP system exhibited good consistency and stability, which was observed over the entire 20 s recording period.

Two different types of sensor-delivery platforms were utilized in this study. The sensor attachment to an IOL takes advantage of well-developed existing cataract surgery and IOL delivery methods, while sensors mounted onto thin silicone haptics for fixation have the advantage of requiring a smaller corneal incision and no further surgical interventions. As a result, sensors could be inserted in 5 min instead of requiring 15 min for invasive lens extraction and IOL insertion. The silicone-haptic approach provided an additional benefit of decreasing the distance between the corneal surface and the sensor from 3–4 mm to 1–2 mm, and this reduction in distance decreased the attenuation through the aqueous humor and the light dispersion caused by the curvature of the cornea. As a result, the SNR of the optical readout increased from 12 to 15 dB, which was comparable to the SNR of 16 dB observed during the bench testing. Lastly, if nanodot-enhanced IOP sensors are eventually used in the clinical setting, sensors mounted on silicone haptics can potentially be implanted in all glaucoma patients in addition to those undergoing lens extraction and IOL placement.

Sensors attached onto the IOLs were implanted using standard surgical techniques for lens phacoemulsification and IOL implantation. This surgical procedure by itself, independent of an attached sensor, can cause corneal edema and a substantial inflammatory tissue reaction in the rabbit eyes, particularly during the first 1–2 weeks after surgery. As the ocular conditions during the first two post-operative weeks after IOL implantation were suboptimal for capturing sensor optical resonance signals, we primarily utilized the sensors attached onto IOLs for longer-term studies beyond 1 month after implantation. On the other hand, sensors mounted on silicone haptics did not elicit any noticeable inflammatory reaction in the anterior chamber after implantation, and the ocular recovery was excellent even on the second day after surgery with only mild incisional edema. Tissue buildup following a 4-month implantation period was noted to be minimal with no evidence of toxicity. As of the manuscript submission date, all of the sensors (*n*=5) remained functional and operational. We will continue to investigate their *in vivo* performance until we have a device failure. Our previous work revealed that the use of nanoscale textured surfaces such as black-silicon on implants can greatly improve the anti-biofouling properties, suggesting an effective way to improve biocompatibility of the sensors^87^. A more detailed histological examination of the sensors will be performed at the end of the study when they are retrieved from the rabbits for re-characterization in the pressure test chamber.

The current version of the nanodot-enhanced IOP sensor is almost an order of magnitude smaller than sensors based on the LC coupling, and further miniaturization is possible. The vertical and lateral dimensions of the sensors can be reduced to 50 and 200 μm, respectively, by using a thinner silicon-on-insulator wafer for fabrication and employing smaller diaphragm designs without degrading the sensor’s mechanical properties. Currently, the sensor size is dictated by the minimum bonding area required to achieve a reliable water-proof seal between the top and bottom structures, and this can be overcome by improving the fabrication or packaging technologies.

In summary, the IOP-monitoring system provides a battery-free operation, invisible interrogation, and convenient and rapid IOP measurements. The implantable IOP sensor is three orders of magnitude smaller than commercially available devices^48,65^, minimizing the risks associated with implantation procedures as well as post surgery and long-term complications. We profiled dynamic changes of IOP in anesthetized rabbits for short-term (1 h) and in awake rabbits for long-term (4.5 months) periods, with an average accuracy of 0.29 mm Hg at a rate of 10 Hz over the physiologically interested range of 0–40 mm Hg. With further sensor refinements and detector automation, the system can become a viable choice for patient-initiated IOP monitoring in the home environment and drug-discovery research in labs. Its use will improve glaucoma management and expedite the search for better glaucoma treatment.
