# Supplementary material for: A microscale optical implant for continuous in vivo monitoring of intraocular pressure
Source: Microsyst Nanoeng. 2017 Dec 18;3:17057. doi: 10.1038/micronano.2017.57 (PMC6445001; doi:10.1038/micronano.2017.57)
Supplement: Supplementary Information [file micronano201757-s1.docx]

**Supplementary file**

A microscale optical implant for continuous *in-vivo* monitoring of intraocular pressure

Jeong Oen Lee^1,2^, Haeri Park^1^, Juan Du^3^, Ashwin Balakrishna^2^, Oliver Chen^2^, David Sretavan^3, 4*^, and Hyuck Choo^1,2*^

- **Fig. S1: Au Nanodot Arrays for Cavity-Resonance Optimization**
- **Fig. S2: Optimization of the Flexible Si_3_N_4_ Membrane Thickness in the NIR Range**
- **Fig. S3: Membrane Deflection Model for Sensor Design and IOP Analysis**
- **Custom-Built Optical Detector**
- **Fig. S4: Sensor Characterization inside a Controlled Pressure Chamber**
- **Fig. S5. IOP-Determination Process**
- **Surgical Procedures**
- **Fig. S6:** ***In Vivo* Intraocular Pressure Monitoring before and after Intravitreal Injection**
- **Fig. S7: Pressure-Sensing Resolution of the IOP Monitoring System**
- **Fig. S8: Angle-resolved Sensor Characterization**

| ^1^Department of Medical Engineering, California Institute of Technology, Pasadena, CA 91106, USA; ^2^Department of Electrical Engineering, California Institute of Technology, Pasadena, CA 91106, USA; ^3^Department of Ophthalmology, University of California San Francisco, San Francisco, CA 94143, USA; ^4^Department of Physiology, University of California San Francisco, San Francisco, CA 94143, USA Correspondence: David Sretavan ([david.sretavan@ucsf.edu)](mailto:david.sretavan@ucsf.edu)), Hyuck Choo ([hchoo@caltech.edu)](mailto:hchoo@caltech.edu)) |
| --- |

**Au Nanodot Arrays for Cavity Resonance Optimization**

The signal-to-noise ratio (SNR) of the sensor is greatest when the peak-to-valley amplitude of the resonance spectrum is maximized. This amplitude maximization would result from matching the reflectivities of the top and bottom surfaces that formed the optical cavity. In order to accomplish this, we embedded an Au-nanodot array on the surface of the Si_3_N_4_ membrane and adjusted its reflectivity by varying the array parameters (**Fig. S1a**). The optimal design of the Au-nanodot array was determined using finite-difference-time-domain (FDTD) simulation studies and corroborated by experimental measurements. A number of Au nanodot arrays of different dot diameters -- 400, 450, 500, 550, 600, 650, 700, 750, and 800 nm – were tested while keeping the array pitch size at 1000 nm (**Fig. S1b**). The dot height (Au thickness) was set at 100 nm. The optical constant of gold was obtained from Johnson and Christy (550 - 950 nm) ^85^. A uniform mesh size of 5 nm was used for the deposited gold dots in all *x*, *y*, and *z* directions. Perfectly matched layer (PML) boundary conditions for the *z* direction, and Bloch boundary conditions for both *x* and *y* directions of the simulation region were used to calculate the wavelength-dependent reflectance spectra (dispersion diagram). **Figures S1c-e** show the experimentally obtained results along with the simulated reflectivities (**Fig. S1d**).


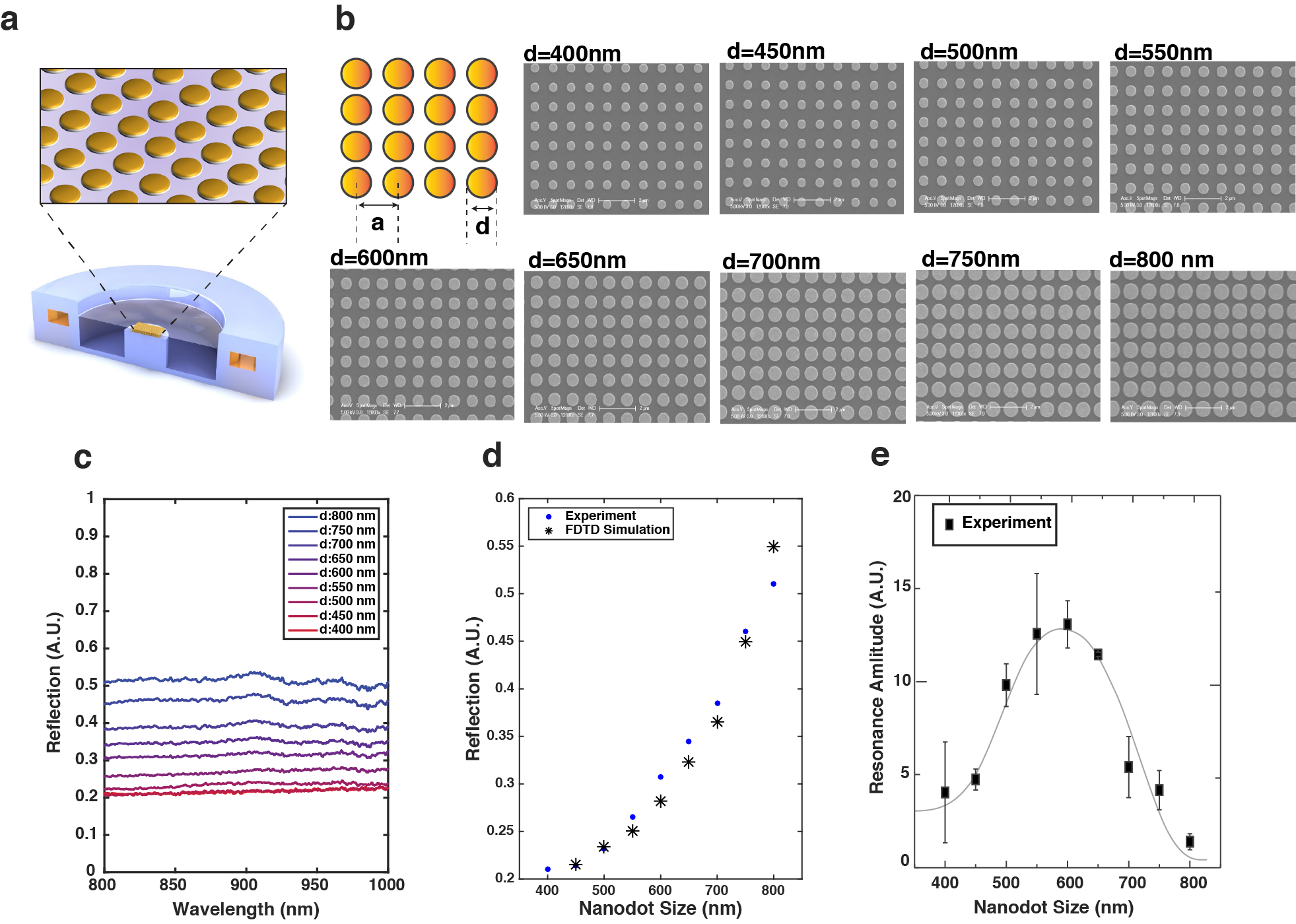


**Figure S1. Au-nanodot array designs for cavity resonance optimization. (a)** A schematic illustration of the Au-nanodot array embedded in the Si_3_N_4_ membrane. **(b)** SEM images of the Au-nanodot arrays with nine different diameters (400, 450, 500, 550, 600, 650, 700, 750, and 800 nm) spaced at an array-pitch size of 1000 nm. The height of the individual dots, which is the thickness of the gold, is 100 nm. **(c)** The reflection measurements as a function of the Au-dot diameters. The dot arrays exhibit almost wavelength-independent reflectivities in the wavelength range of interests. **(d)** The comparison between experimentally measured reflectivities *vs*. FDTD-simulation results for nine different Au-dot diameters. **(e)** Experimentally characterized resonance amplitudes as a function of Au-nanodot diameters; the optimal diameter was found to be 600 nm.

**Optimization of the Flexible Si_3_N_4_ Membrane Thickness**

The final resonance spectrum captured in sensor detection is composed of two separate resonances: one from the thickness of the Si_3_N_4_ membrane and the other from the optical cavity between the bottom surface of the Si_3_N_4_ membrane and the top surface of the Si substrate. The resonance from the Si_3_N_4_ membrane defines the outer envelope of a lower frequency (**Fig. S2a-b**, black dashed lines) while that of the cavity forms the inner resonance component with a higher frequency (red solid lines). In order to maximize the resonance amplitude, which was defined as a peak-to-valley height of the resonance spectrum, we optimized the Si_3_N_4_ membrane thickness in the wavelength range of interest so that the maximum of its resonance envelope would be centered in the range of 700-1100 nm. Using an analytical optical model and FDTD simulations, we found that the optimal thickness of the silicon-nitride membrane was 0.3 *μ*m.


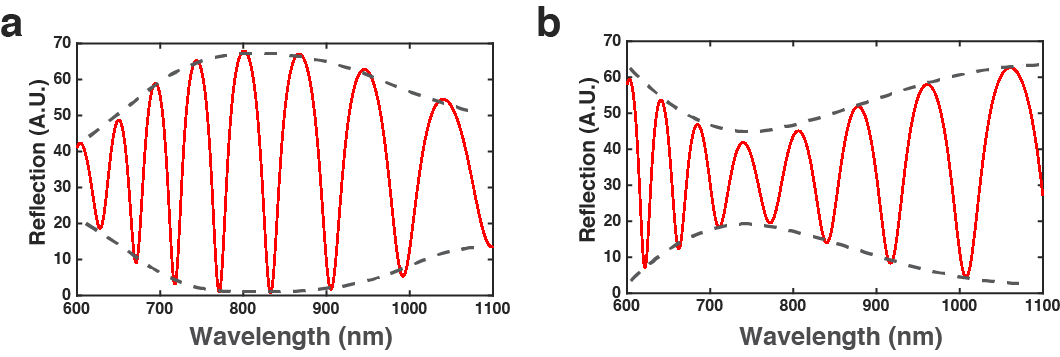


**Figure S2. Optimizing the Si_3_N_4_ membrane thickness in the NIR range.** **(a)** The calculated reflection spectrum when the Si_3_N_4_ membrane thickness is 300 nm; the peak-to-valley height is maximized in the wavelength range of interest. **(b)** The calculated reflection spectrum when the Si_3_N_4_ membrane thickness is 190 nm; this would be the worst case that lowers the amplitude and degrades the remote readout performance.

**Membrane-Deflection Model for Sensor Design and IOP Determination**

In an analytical model, we used a freely deformable diaphragm with fixed edges for the boundary condition. To reduce the computation time, the actual design of the pressure sensor was simplified to a single circular-shaped diaphragm model, whose bottom surface experiences 1 atm (the pressure inside the cavity) while the top surface experiences varying pressures (IOP). A free-tetrahedral mesh was used to define the three-dimensional finite elements to improve the modeling accuracy. The following values were used for the mechanical properties of Si_3_N_4_: Young’s modulus of 265 GPa and Poisson’s ratio of 0.27.


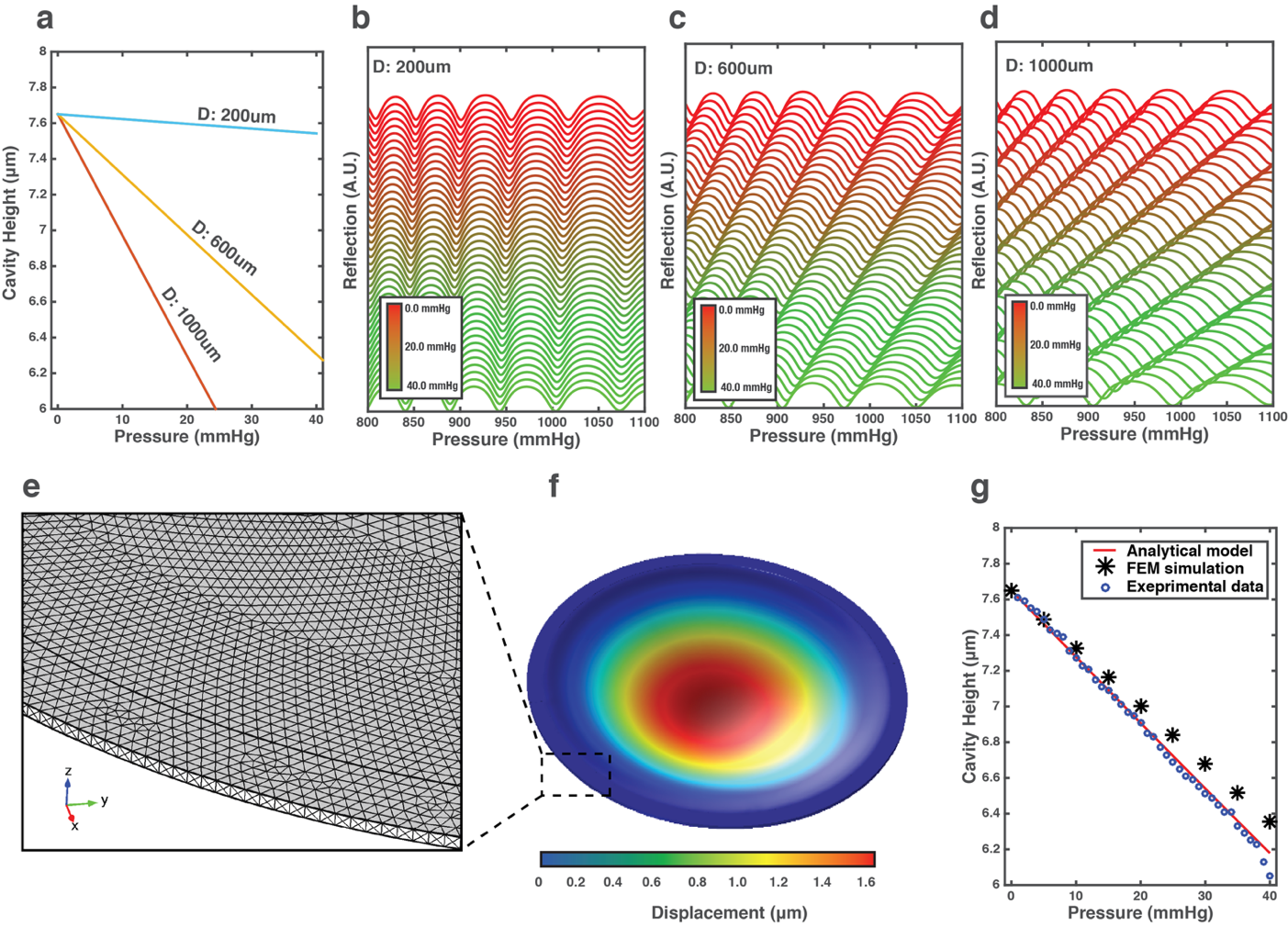


**Figure S3. Membrane deflection model for sensor design and IOP analysis. (a)** The calculated magnitude of the membrane deflection as a function of pressure change for three different diameters of Si_3_N_4_ membranes: 200, 600, and 1000 *μ*m. The thickness of each membrane is 300 nm. In the pressure range of interest (0 – 40 mmHg), all membranes deform linearly. The 600-*μ*m membrane provides complete coverage of the pressure range as well as good sensitivity. **(b)** The calculated resonance shift in the 200-*μ*m diameter membrane as a function of pressure. The spectrum shift is very small because the membrane is too stiff. **(c)** The 600-*μ*m membrane exhibits a linear shift of resonance over the entire pressure range of interest. **(d)** The spectrum shift in the 1000-*μ*m diameter membrane. The coverage of the pressure range is incomplete, and as a result, there are too many overlapping peaks and valleys, making IOP determinations challenging. **(e)** The mesh generation for finite element-method (FEM) simulation. The computational domain was meshed using a tetrahedral grid. The mesh size used in this model ranged from 100 to 500 nm. **(f)** The 3D diagram of the membrane deformation from the FEM simulation. The maximum deformation of the 600-*μ*m membrane was predicted to be 1.4 *μ*m at 40 mmHg. **(g)** The comparison of the cavity heights predicted by the mechanical model, by FEM simulations, and from the experimental measurements over the pressure range of interest; the experimental results and the values predicted by the mechanical model given in the reference matched very well. This model was also used to back-calculate the IOP from the measured spectra.

**Custom-Built Optical Detector**

A schematic of the optical resonance detector setup mainly used for table-top measurements is shown in **Fig. 2a**. An infinity-corrected microscope objective (20x, Plan, Apo, Infinity Corrected, Long Working Distance, Mitutotyo, Kawasaki, Japan) with a numerical aperture (NA) of 0.42 was used to image and focus on the SiN membrane surface of the sensor. The image of the sensor was captured using a USB camera (STC-MB133USB, Sentech Co., Atsugi, Japan) and displayed on a computer screen. A XYZ translation stage (PT3, Thorlabs Inc., Newton, NJ, USA) was used to adjust the axial position of the field of view with micrometer precision. The interrogating light was delivered from a broadband light source (OSL1 High-Intensity Fiber Light Source, Thorlabs Inc., Newton, NJ, USA) through a polarizer, a dichroic beam splitter (pass: NIR; reflect: VIS), a quarter wave plate, the microscope objective and finally to the sensor under study. This polarizing optical configuration effectively eliminated the ambient optical noise from our measurements and passed only the properly polarized interrogating light. The reflection from the sensor was collected through the same optical path and relayed to an off-the-shelf, commercially available mini VIS-NIR spectrometer (Maya 2000 Pro, 780 nm-1200 nm, resolution of 0.22 nm, Ocean Optics, Dunedin, FL, USA). Readout was performed at a distance of 3-5 cm, and the theory predicts that the readout distance could be extended up to 80 cm.

**Sensor Characterization in a Controlled Pressure Chamber**


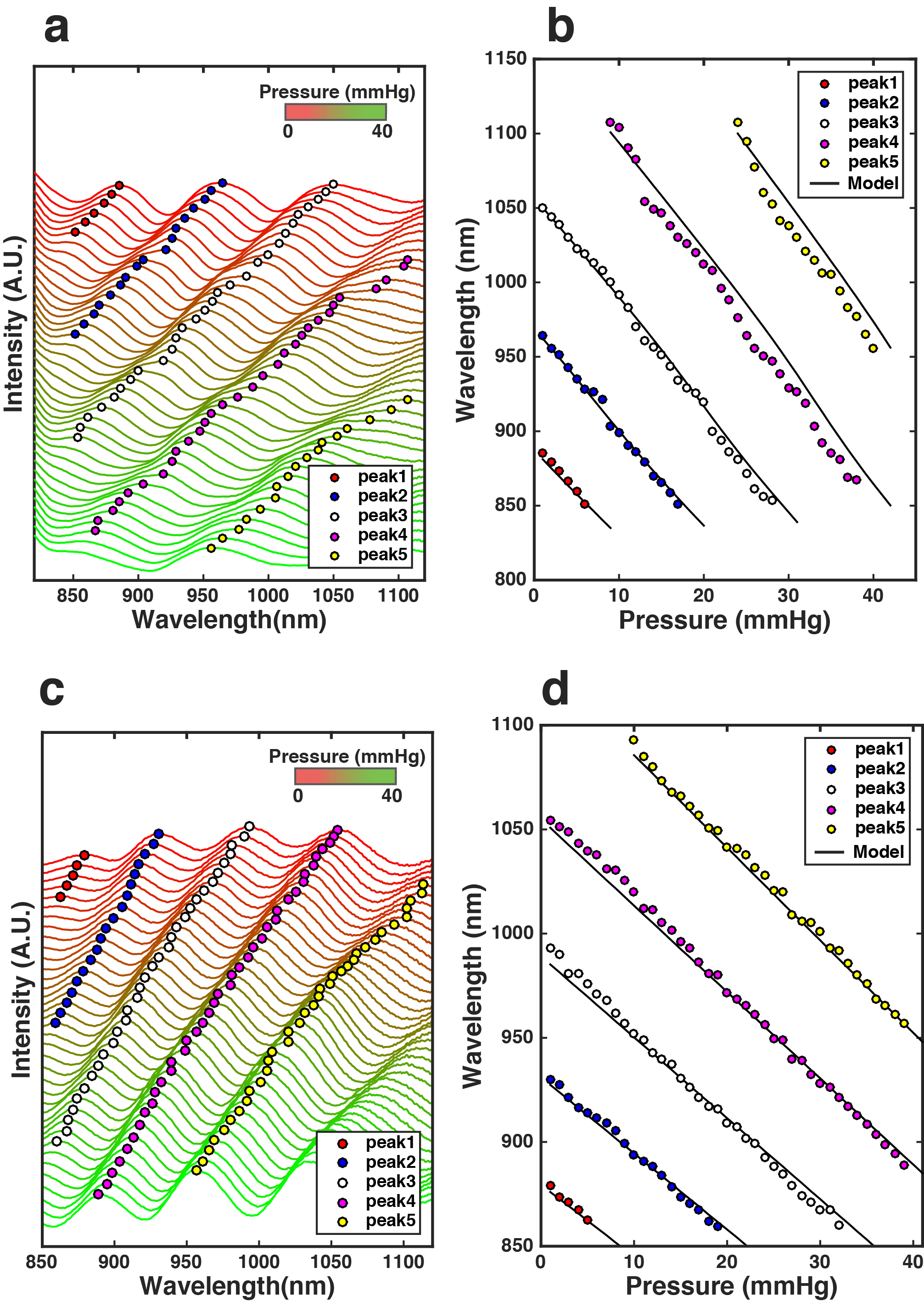


**Figure S4. Characterization of IOP sensors in a controlled pressure chamber.** **(a)** The experimentally measured spectra of the sensor shown in Figure 2 and the automatically identified peaks (indicated by circles) using our optomechanical algorithm. **(b)** The theoretically predicted and experimentally obtained spectral peak predictions by the optomechanical model (black solid lines) and the experimentally obtained peak locations (circles) *vs*. pressure change. These values match very closely. **(c)** The empirical spectra and the identified peak locations of the sensor shown in Figure 3. **(d)** The extracted peak locations (circles) and the theoretical predictions (black solid lines) match very closely.

**IOP-Determination Process**

**
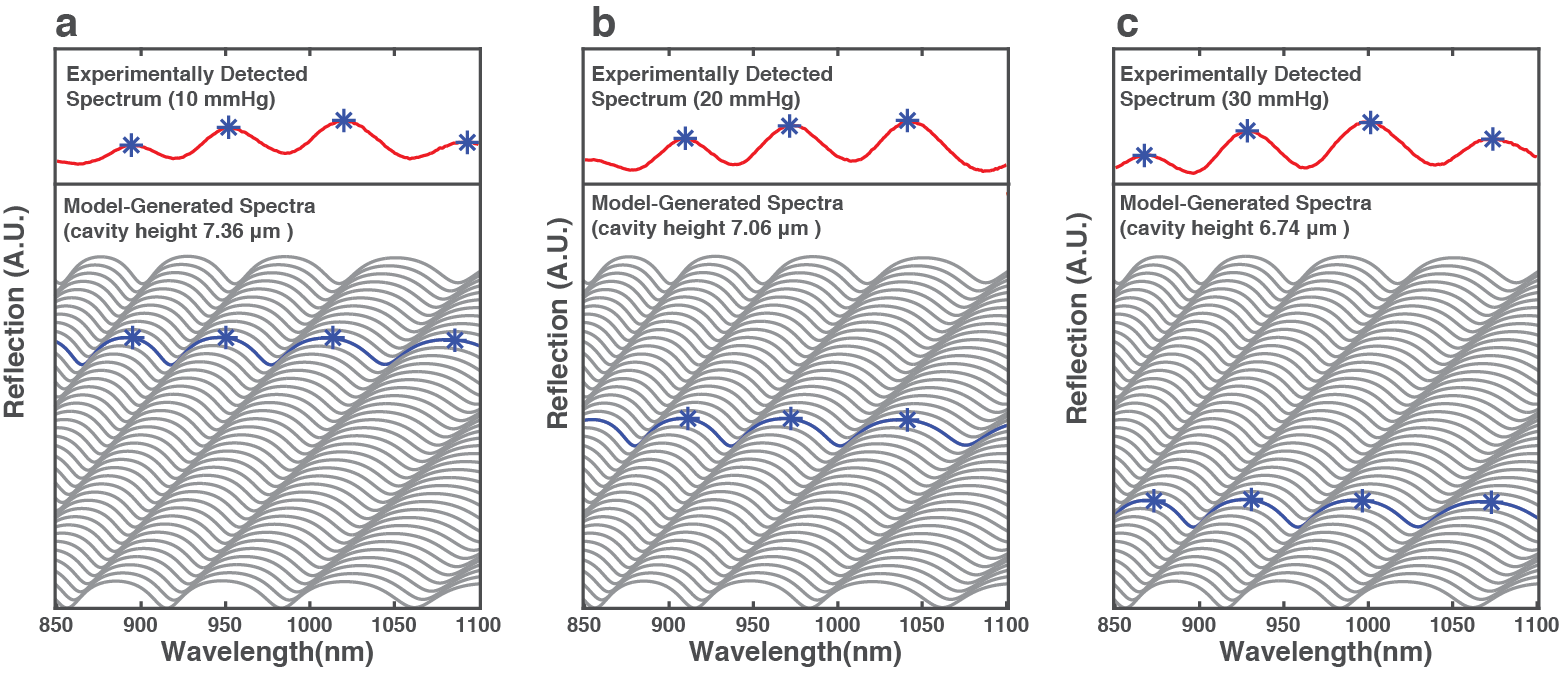
**

**Figure S5. IOP-determination process.** **(a)** The measured reflection spectrum from the sensor at 10 mmHg (top) and a set of calculated reflection spectra in the pressure range of interest (bottom). Based on the locations of the extrema (peaks), our algorithm automatically searches through the model-generated spectra and finds the best matching one for the experimentally detected spectrum. **(b)** An automated IOP identification at 20 mmHg. **(c)** An automated IOP identification at 30 mmHg.

**Data Processing and IOP Determination**

The procedure for determining the IOP from the raw spectra was composed of four stages: (1) de-noising and low pass filtering of the raw spectra, (2) spectral feature recognition, (3) fitting with a theoretical resonance spectrum, and (4) determining the IOP based on the optomechanical model (OMM) (see below). We first pre-processed the captured spectra through a de-noising filter to reveal the peaks and valleys or extrema in the resonance spectra. Next, the locations of the peaks and the valleys were automatically identified using an extrema-matching technique (**Fig. S4 and** **Fig. S5**). One can observe the highly linear shift in the extrema locations as the pressure is increased.

**Surgical Procedures**

**Sensor Attachment to Delivery Platforms**

The nanodot-enhanced sensors were attached to either an IOL or to silicone haptics for delivery and placement into the eye. A cavity of approximately 1 mm in diameter and 0.5 mm in depth was mechanically cut into the IOL. Sensors were placed into this cavity using medical grade UV adhesive (Loctite 3321, Loctite Corp. Rocky Hill, CT, USA) with the deformable membrane side facing outwards from the IOL cavity. The silicone haptics were manually fabricated from medical grade silicone membrane (125 μm thick) (BioPlexus, Ventura, CA, USA) into a barbell shape approximately 2.5 mm in width and 12.5 mm in length. The sensor was attached to the silicone haptics with medical grade silicone adhesive (Med-1000 RTV silicone adhesive, Nusil Technology, Carpenteria, CA, USA). The sensor and haptics were sterilized using 70% alcohol for 15 minutes and then rinsed with sterile 0.9% saline solution prior to use.

**Animals**

The New Zealand White rabbits (females 2.5-3.2 kg in weight) were obtained from Western Oregon Rabbit Company (Philomath, OR. USA) and housed in an USDA approved facility at UCSF. The use of animals in this study adhered to the Association for Research in Vision & Ophthalmology Statement for the Use of Animals in Ophthalmic and Vision Research. The experimental protocol in this study has been approved by the Institutional Animal Care and Use Committee (IACUC) of the University of California San Francisco. Rabbits were used in an observational study of *in vivo* sensor performance. The results reported here were broadly representative of the 5 sensors implanted and studied up to 4.5 months. No statistical analysis was performed to compare results from different rabbits. No randomization of animals was performed and no masking of the investigators was utilized.

**Implantation of Sensors Attached to Intraocular Lenses**

Dilating eyedrops (phenephrine 2.5% and cyclopentolate 1%) were applied to the eye intended for surgery once every 5 minutes for a total of three times. Ketamine (35mg/kg) and xylazine (3mg/kg) were then intramuscularly injected. The animal was transferred to the surgical table and placed under isoflurane anesthesia. The heart rate, breathing rate, core temperature, and O_2_ saturation were monitored throughout surgery. Topical proparacaine hydrochloride (0.5%) was applied to the eye, followed by 1 drop of 5% povidone-iodine. A 1-mm sideport incision was made followed by the injection of an ophthalmic viscosurgical device (OVD) (Provisc, Alcon, Fort Worth, TX, USA) into the anterior chamber. A 3.0-mm primary corneal incision was created, and capsulorhexis forceps were introduced to perform a 4.5-mm capsulotomy. Following hydrodissection, the native lens was subjected to phacoemulsification (Alcon Infiniti Phacoemulsification System, Alcon, Fort Worth, TX, USA), and irrigation and aspiration were performed for cortical cleanup using 0.9% saline with 1:1000 epinephrine and 10 IU/mL heparin. A one-piece acrylic IOL (Acrysof SN60 WF, Alcon, Fort Worth, TX, USA) with a sensor attached was inserted into the recommended IOL cartridge and loaded into the IOL inserter according to the manufacturer’s instructions. The folded IOL with the sensor was then inserted into the capsular bag, and the IOL position was adjusted as needed. The OVD was then removed by irrigation, and one 10-0 suture was placed to close the primary incision. Cefazolin antibiotic was applied sub-conjunctivally and a neomyxin / dexamethasone ointment was applied to the eye. The rabbit was then removed from anesthesia and allowed to revive before being returned to the housing area.

**Implantation of Sensors with Silicone Haptics**

The pre-surgical preparation, anesthesia, and monitoring of rabbits was as described above for animals with IOL / sensor implantation. A 2.8-mm corneal incision was made and OVD injected into the anterior chamber. The sensor with silicone haptics was folded onto itself, grasped with a pair of forceps, and introduced through the corneal incision. The haptics were allowed to unfold spontaneously and positioned into the iridocorneal angles for fixation. The OVD was then removed by irrigation, and a 10-0 suture was placed to close the incision as needed. A subconjunctival antibiotic and an antibiotic / steroid ointment were applied as described above.

***In Vivo* IOP Monitoring after Intravitreal Injection**

**
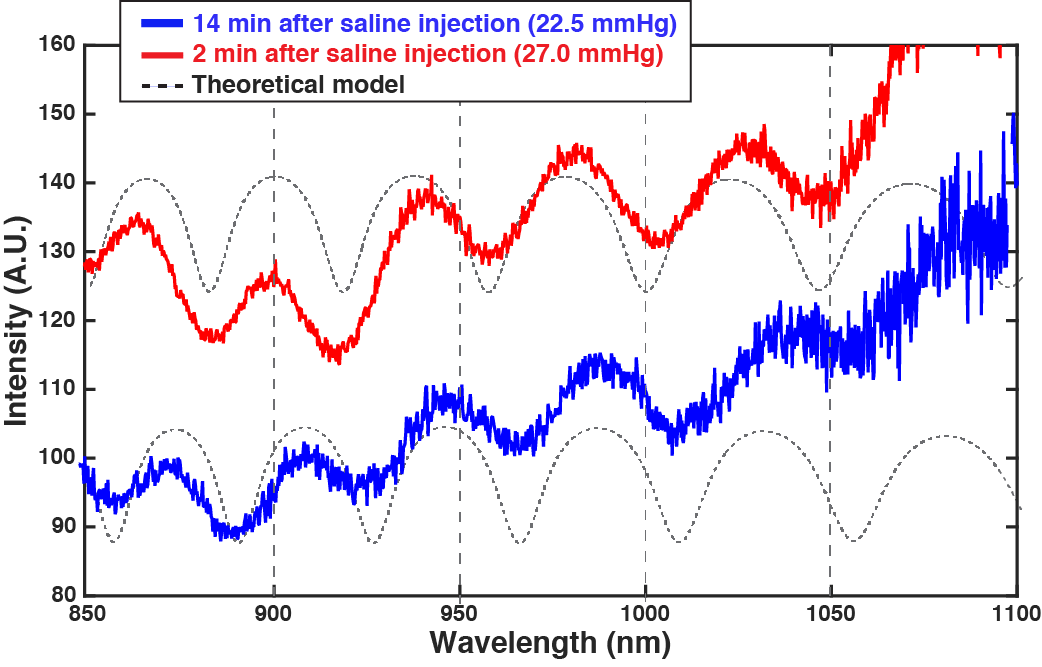
**

**Figure S6.** ***In Vivo* IOP monitoring after intravitreal injection.** The spectrum measurement after the intravitreal injection of saline. The red and blue lines indicate the measured spectrum after the saline injections at 2 min and 14 min, respectively. The dashed black lines correspond to the best-fitting curve using the optomechanical model. The sensor-derived IOP values decreased from 27.0 to 22.5 mmHg as time progressed.

**Pressure Resolution of the Nanophotonic Sensor IOP-Monitoring System**


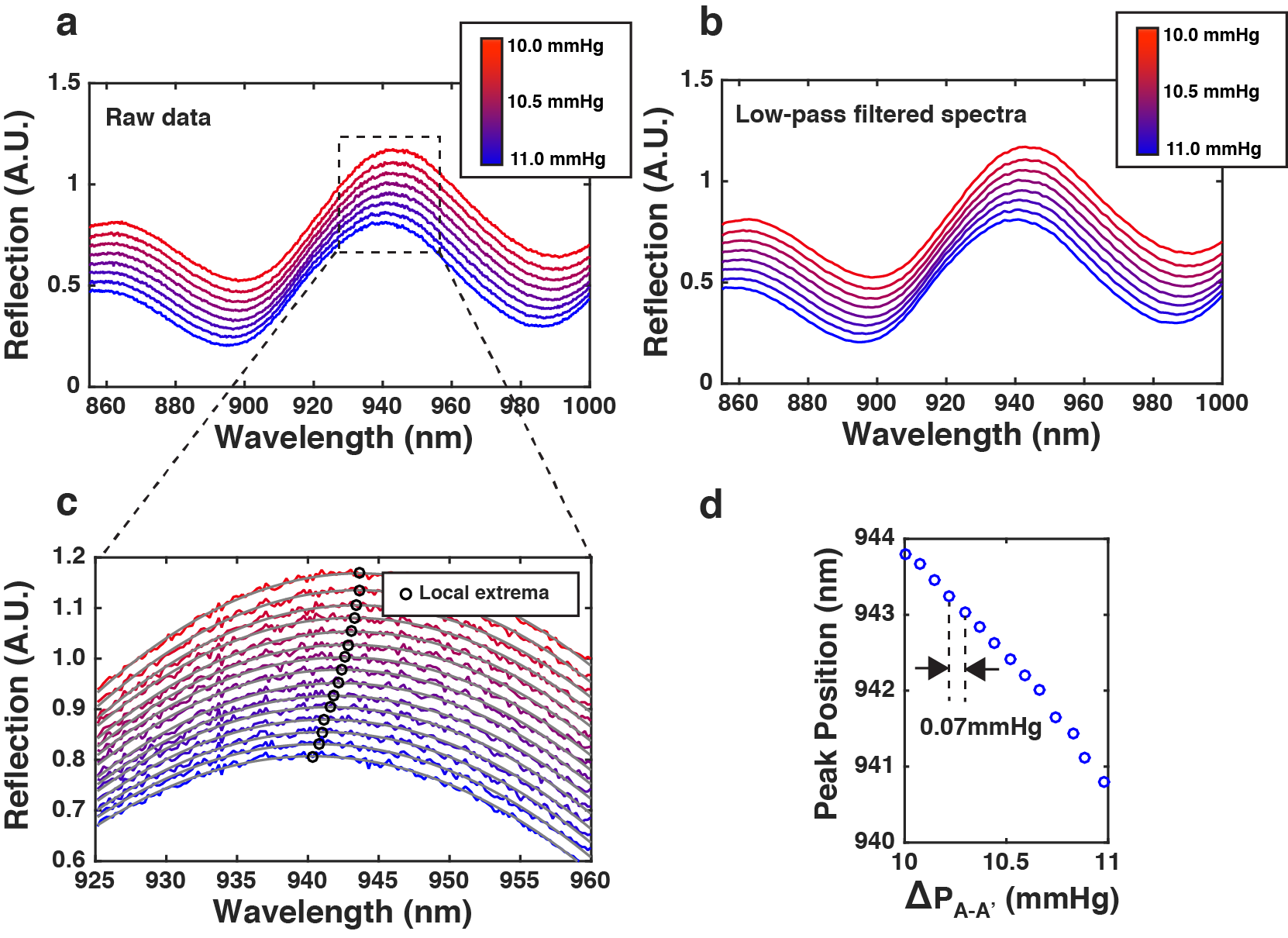


**Figure S7. Pressure resolution of the IOP-monitoring system.** **(a)** The measured reflection spectra from the sensor in the pressure range of 10 to 11 mmHg. Using a microtranslational stage, we adjusted the water height at 0.1 mm resolution to precisely control the hydrostatic pressure inside the pressure-controlled chamber. **(b)** The removal of high frequency noise in the measured spectra using a low-pass filter. **(c)** The automated identification of local extrema. **(d)** The pressure mapping: peak locations *vs.* corresponding pressures. The minimum sensing resolution was 0.07 mmHg, which corresponds to the water height change of 1.0 mm.

**Angle-resolved Sensor Characterization**


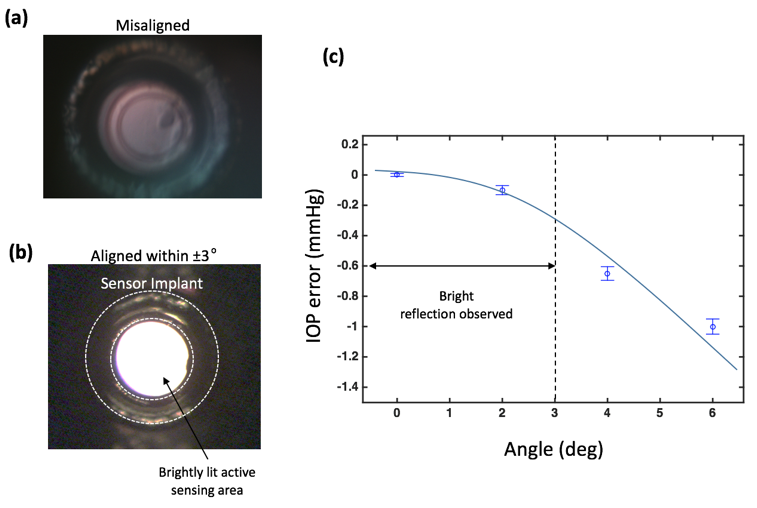


**Figure S8. Angle-resolved sensor characterization.** **(a)** The USB camera image of the sensor: the misalignment between the sensor and the detector is over 3°. **(b)** A bright reflection is observed when the misalignment becomes less than about 3°. **(c)** Experimentally characterized IOP identification error vs. measurement angle: When the alignment is within ± 3°, a bright reflection from the sensor is observed through the USB camera, and the error remains below 0.3 mmHg.
